# High content analysis identifies unique morphological features of reprogrammed cardiomyocytes

**DOI:** 10.1038/s41598-018-19539-z

**Published:** 2018-01-19

**Authors:** Matthew D. Sutcliffe, Philip M. Tan, Antonio Fernandez-Perez, Young-Jae Nam, Nikhil V. Munshi, Jeffrey J. Saucerman

**Affiliations:** 1 Department of Biomedical Engineering, University of Virginia, Charlottesville, VA 22908 USA; 2 Department of Internal Medicine, Division of Cardiology, UT Southwestern Medical Center, Dallas, TX 75390 USA; 3 Department of Medicine, Division of Cardiovascular Medicine, Vanderbilt University Medical Center, Nashville, TN 37232 USA; 4 Department of Molecular Biology, UT Southwestern Medical Center, Dallas, TX 75390 USA; 5 McDermott Center for Human Growth and Development, UT Southwestern Medical Center, Dallas, TX 75390 USA; 6 Hamon Center for Regenerative Science and Medicine, UT Southwestern Medical Center, Dallas, TX 75390 USA

## Abstract

Direct reprogramming of fibroblasts into cardiomyocytes is a promising approach for cardiac regeneration but still faces challenges in efficiently generating mature cardiomyocytes. Systematic optimization of reprogramming protocols requires scalable, objective methods to assess cellular phenotype beyond what is captured by transcriptional signatures alone. To address this question, we automatically segmented reprogrammed cardiomyocytes from immunofluorescence images and analyzed cell morphology. We also introduce a method to quantify sarcomere structure using Haralick texture features, called SarcOmere Texture Analysis (SOTA). We show that induced cardiac-like myocytes (iCLMs) are highly variable in expression of cardiomyocyte markers, producing subtypes that are not typically seen *in vivo*. Compared to neonatal mouse cardiomyocytes, iCLMs have more variable cell size and shape, have less organized sarcomere structure, and demonstrate reduced sarcomere length. Taken together, these results indicate that traditional methods of assessing cardiomyocyte reprogramming by quantifying induction of cardiomyocyte marker proteins may not be sufficient to predict functionality. The automated image analysis methods described in this study may enable more systematic approaches for improving reprogramming techniques above and beyond existing algorithms that rely heavily on transcriptome profiling.

## Introduction

Cardiomyocytes have limited regenerative capacity in the adult heart, and following a myocardial infarction many cardiomyocytes are irreversibly lost^1^. In response, activated fibroblasts proliferate, migrate into the injured area, and deposit collagen and other extracellular matrix proteins^2,3^. A scar forms and, over time, the contractile function of the heart weakens, leading to congestive heart failure. To contend with this growing clinical problem, methods for generating new cardiomyocytes are greatly needed. For example, pluripotent stem cells (i.e. ESCs or iPSCs) can be expanded and differentiated *ex vivo* prior to transplantation. Although this approach has shown promise in large animal models^4,5^ and tumors have not been observed to date, the use of pluripotent stem cells raises concerns of teratogenicity. An alternative therapeutic strategy that bypasses the concerns of the cell transplantation approach involves direct conversion of fibroblasts into functional cardiomyocytes^6,7^. This approach is particularly attractive, since it can be accomplished in activated fibroblasts *in situ* to convert them into cardiomyocytes rather than form scar tissue^8,9^.

Direct reprogramming involves transduction of various combinations of transcription factors that typically consist of key developmental regulators. The most commonly described transcription factor combination for induced cardiac-like myocyte (iCLM) reprogramming includes Gata4 (G), Mef2c (M), and Tbx5 (T), with or without Hand2 (H)^8,10^. GMT and GHMT both convert fibroblasts into functional cardiomyocytes *in vitro* and *in vivo*^9,11^. Interestingly, *in vivo* reprogramming is substantially more efficient than *in vitro* reprogramming, suggesting that various aspects of the endogenous milieu are likely to influence reprogramming efficacy. Nevertheless, the precise mechanisms responsible for reprogramming, and the ideal transcription factor combinations required to produce mature subtype-specific cardiomyocytes remain unclear. One major hurdle that has slowed progress in this field is the lack of objective and quantitative measures of cardiomyocyte reprogramming. We recently found that GHMT generates all three cardiomyocyte subtypes (i.e. atrial, ventricular, and pacemaker) but with low efficiency due in part to incomplete sarcomere formation^12^. Thus, we sought to develop an unbiased algorithm for assessing cardiomyocyte subtype and sarcomere structure in directly reprogrammed fibroblasts.

Automated image processing algorithms to extract morphological and textural information provide objective and quantitative methods to analyze cells. These methods may be used to assess the function of induced cardiomyocytes. Cardiomyocytes are composed of bundles of myofibrils, each of which consists of distinct, repeating sarcomeres. Thus, the sarcomere is the basic force-generating unit of striated muscle. Sarcomeres are composed of myosin and actin, the two components of cross-bridge formation, and Z-lines, which are protein complexes defining the edges of the sarcomeres. It is intuitive that clearer sarcomere structure, as indicated by Z-line structure in the α-actinin stain, is correlated with cardiomyocyte functionality. We have previously shown that sarcomere organization is a prerequisite for reprogrammed cardiomyocytes to spontaneously contract, a well-established parameter of functionality^12^. To usefully reprogram cardiomyocytes, therefore, careful attention must be given to both cell morphology and contractility.

To quantify and thus compare iCLMs with neonatal mouse cardiomyocytes, we have developed a fully automated method of segmenting cells from multi-channel immunofluorescence images and used this to analyze their morphology. To quantify sarcomere structure, we developed a method, based on offset distance–angle distributions of Haralick texture features, called SarcOmere Texture Analysis (SOTA). Using this method, we found that current methods of direct reprogramming generate cardiomyocytes with less organized sarcomeres, shorter sarcomere lengths, as well as an apparent lack of coordination between cellular elongation and sarcomere alignment. These new automated image analysis methods may facilitate quantitative screening of experimental protocols that further enhance the efficiency and fidelity of cardiomyocyte reprogramming.

## Results

### Automated subtype classification and morphological analysis of induced cardiac-like myocytes

We previously developed algorithms for automated cell segmentation and morphological analysis of primary neonatal cardiomyocytes based on a combination of DAPI and α-actinin^13^. Here, we extended that method to include multiple cardiomyocyte markers (α-actinin, Hcn4, and Nppa) that distinguish the diverse cell subtypes that arise from cell reprogramming by GHMT transduction. Hcn4 is an ion channel predominantly expressed in pacemaker cardiomyocytes and Nppa is a perinuclear marker of atrial cardiomyocytes^12^. The heterogeneous expression of these three markers in reprogrammed cardiomyocytes required a more flexible method for systematically identifying and classifying nuclei, cell borders, and cell subtypes.

Neonatal mouse atrial cardiomyocytes (CMs) and GHMT-reprogrammed cells were fixed and stained for DAPI, α-actinin, Hcn4-GFP, and Nppa. Nuclei were first identified by thresholding the processed DAPI channel using Otsu’s method^14^. The α-actinin and Hcn4-GFP images were similarly thresholded by Otsu’s method. Nuclei that were fully within α-actinin^+^ or Hcn4-GFP^+^ regions were classified as positive for those respective cardiomyocyte markers. Nppa classification was based on the 90^th^ percentile intensity within the perinuclear region, defined as the area extending 8 pixels (1.25 μm) from the nuclear boundary. Nuclei of the same classification group that were within 25 pixels (3.91 μm) of one another were joined and assumed to be part of a bi-nucleated cell, as validated previously^13^.

**Figure 1:**
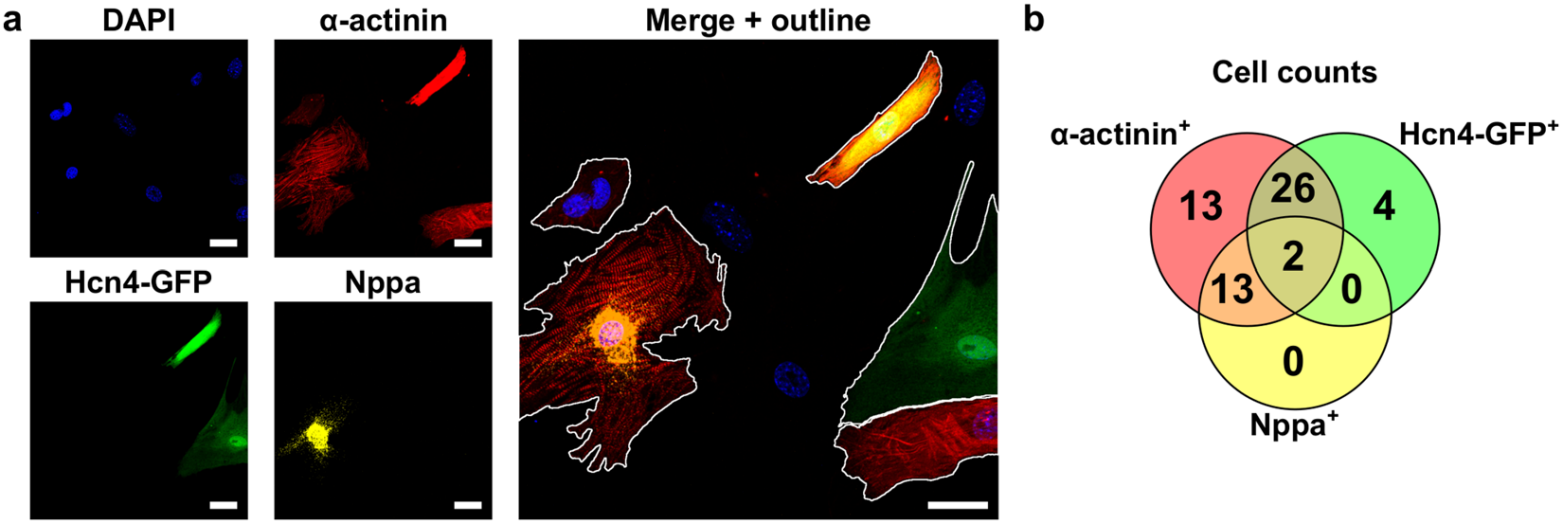
Automated cell segmentation identifies induced cardiac-like myocytes (iCLMs) with heterogeneous marker expression. (**a**) Mouse embryonic fibroblasts were isolated from E13.5 mouse embryos and reprogrammed by transfecting with retroviral constructs of GHMT. Cells were examined by immunofluorescence for expression of cardiac markers α-actinin, Hcn4-GFP, and Nppa. The merged image is shown on right with automated cell segmentation outlines in white. Scale bar = 20 μm. (**b**) Venn diagram showing the distribution of cardiac marker expression among iCLMs. Only iCLMs that expressed at least one marker are shown. Most cells (132) were not positive for any cardiac marker.

Cell boundaries were segmented based on sequentially masked images to better distinguish between neighboring cells with distinct α-actinin^+^ and Hcn4-GFP^+^ expression. First, Hcn4-GFP^+^ regions were masked to allow segmentation of α-actinin^+^/Hcn4-GFP^−^ cells. Next, α-actinin^+^ regions were masked to segment α-actinin^−^/Hcn4-GFP^+^ cells. Finally, the inverse of the intersection of these two regions was used to mask a merged α-actinin/Hcn4-GFP image to segment α-actinin^+^/Hcn4^+^ cells. In all cases, segmentation was performed via the watershed method of the gradient-transformed image, which finds regions of maximally changing intensities^15^. An example segmented image is shown in Fig. 1.

Most GHMT-transduced cells were negative for all cardiomyocyte markers, indicating the low efficiency of current reprogramming methods^12^. GHMT-transduced cells were classified as induced cardiac-like myocytes (iCLM) if they expressed α-actinin. Further confirmation of cardiomyocyte induction and maturity was assessed by pericentriolar material 1 (PCM1) staining^16^ (Supplementary Figure S1). The GHMT-transduced cells expressed five out of seven possible combinations of the α-actinin, Hcn4-GFP, and Nppa markers (Fig. 1b). Induced atrial-like cells (α-actinin^+^/Nppa^+^) and induced pacemaker-like cells (α-actinin^+^/Hcn4-GFP^+^) were identified, suggesting that these methods can produce cardiomyocytes similar to the defined phenotypes found *in vivo*. Furthermore, we have previously shown that induced atrial-like cells also express Myl7, an additional atrial cardiomyocyte marker^12^. In addition, several unexpected combinations were found. Some cells were only positive for the Hcn4-GFP marker, and could be incompletely reprogrammed or mis-programmed cells. Although Hcn4 is expressed widely during heart development^17^, we have previously shown that GHMT-transduced fibroblasts do not pass through an Nkx2.5 lineage-positive intermediate^12^. Therefore, it is more likely that α-actinin^−^/Hcn4-GFP^+^ cells represent mis-specification of cell state, as Hcn4 is also expressed in specific regions of the central nervous system, including the cerebellum^18^. Additionally, two cells were identified and manually confirmed to be α-actinin^+^/Hcn4-GFP^+^/Nppa^+^. These cells tended to have more visually distinguishable sarcomeres and may represent so-called transitional cells that surround the sinoatrial node^19^. Finally, several cells were only α-actinin^+^, which may be induced ventricular-like myocytes^12^. While this GHMT method of reprogramming has been shown to generate α-actinin^+^/Myl2^+^ cells, we could not confirm this within our current limitation of visualizing four separate fluorescent channels (DAPI, Hcn4-GFP, Nppa, and α-actinin). However, we have previously shown that Nppa^+^ reprogrammed cells do not express Myl2^12^.

**Figure 2:**
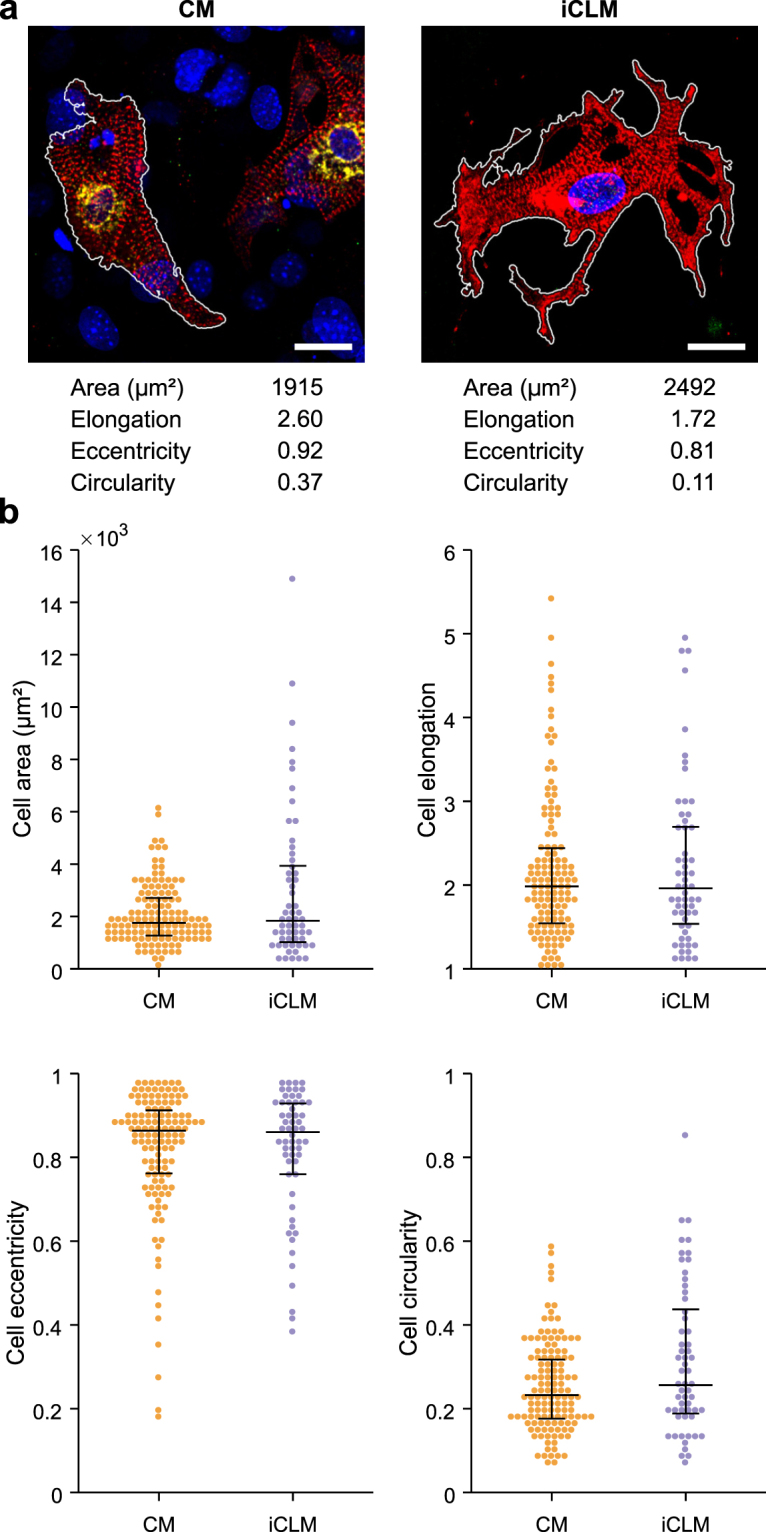
Increased morphological variability of reprogrammed iCLMs compared to endogenous CMs. (**a**) Segmented immunofluorescence images of endogenous CMs (left) and reprogrammed iCLMs (right), with automated morphology measurements for these cells below. Scale bar = 20 μm. (**b**) Population measurements of various morphological features. Black bars represent 25^th^, 50^th^, and 75^th^ percentile.

After automated segmentation, cells were analyzed for area and other morphological characteristics (Fig. 2). iCLMs and CMs had similar median cell areas, however iCLM area was more variable. Indeed, several iCLMs were substantially larger than the range seen for endogenous CMs. We found that most of these larger iCLMs were α-actinin^+^/Hcn4-GFP^+^ (Supplementary Figure S2). Higher variability of iCLMs was also seen in cell circularity. In contrast, eccentricity and elongation, which are related to overall aspect ratios, were similar between iCLMs and CMs, with most cells exhibiting a major/minor axis ratio of about 2:1. This morphological variability suggests that future reprogramming techniques could benefit from improved methods for controlling cardiomyocyte size.

### Quantitative metrics for measuring sarcomere organization

The presence of visually distinguishable sarcomeres is frequently used to indicate cardiomyocyte maturity and functionality^17,20^. Most current methods involve subjectively analyzing sarcomere structure or using Fourier transforms on manually selected rectangular sub-regions to quantify sarcomere organization^21^, potentially introducing bias. To address these limitations, we developed a pixel-based image analysis method for assessing sarcomere structure, called SarcOmere Texture Analysis (SOTA). SOTA utilizes Haralick texture features, which are calculated from the gray level co-occurrence matrix (see Methods), that can be applied to any geometric shape^22,23^. In SOTA, one of 13 Haralick texture features is computed for a range of orientations (0° to 180°) and pixel offset distances, forming an offset distance–angle distribution from which various features of sarcomere structure can be extracted.

**Figure 3:**
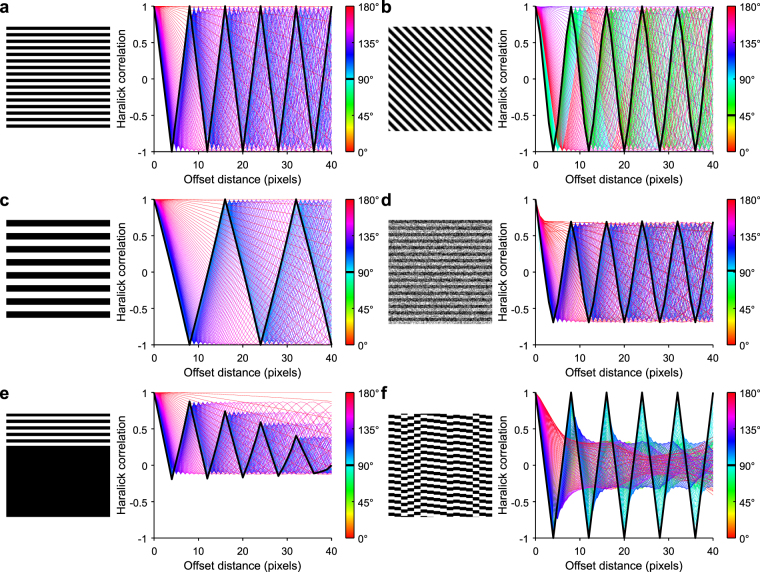
Haralick correlation metric in idealized images. (**a**) Horizontal stripes. (**b**) Diagonal stripes. (**c**) Horizontal stripes of a different frequency. (**d**) Horizontal stripes with noise added. (**e**) Horizontal stripes in one third of the image. (**f**) Bands with random vertical offset.

We first applied SOTA to images with stripes that are representative of idealized sarcomeres (Fig. 3). As shown in Fig. 3a,b, such stripes produced repeated peaks in Haralick correlation, with the greatest magnitude in the direction of the sarcomeres. Increasing sarcomere length spreads these correlation peaks (Fig. 3c), while introducing noise reduces the magnitude of the correlation peaks (Fig. 3d). The rate of decay in correlation peaks in the sarcomere direction is sensitive to the persistence of serially aligned sarcomeres (Fig. 3e), while decay in the longitudinal direction is sensitive to the width of the sarcomere bands (Fig. 3f).

**Figure 4:**
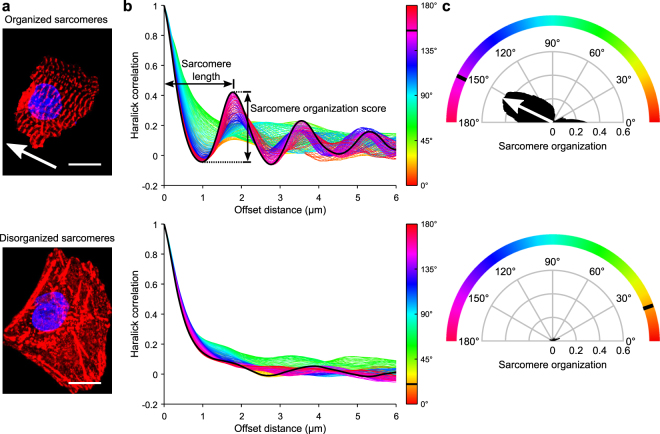
Automated measures of sarcomere organization and sarcomere length. (**a**) Masked immunofluorescence images of neonatal mouse CMs with organized (top) or disorganized (bottom) sarcomeres. Scale bar = 10 μm. (**b**) Haralick correlation metric computed at multiple offset distances and angles to determine sarcomere organization and sarcomere length. Sarcomere organization score is the maximum amplitude of the decaying sinusoidal trace. Sarcomere length is the distance to the first peak. (**c**) Sarcomere organization assessed as a function of angle to assess the primary direction of sarcomere alignment. Arrow points in the direction of sarcomere alignment and is repeated in (**a**). Color bar in middle panel aligns with circumferential color bar in right panel.

We then applied SOTA to representative neonatal mouse CMs that had been subjectively classified as having highly organized or disorganized sarcomeres (Fig. 4). In cells with organized sarcomeres, a characteristic striated pattern is observed in the α-actinin stain. The corresponding Haralick offset distance–angle distributions of real cells decay more rapidly towards zero with increasing offset distance, because such pixels are less likely to be of similar intensity. At the angle corresponding to the fiber direction, this trace develops a decaying sinusoidal pattern, with peaks representing sets of pixels of similar intensity a specified distance apart (Fig. 4b). Biologically, these peaks are indicative of adjacent Z-lines within the cytoskeletal structure. Sarcomere organization is quantified by calculating the maximum peak prominence of all the traces. The angle at which this maximum occurs is the primary sarcomere direction. Sarcomere length is the pixel offset distance of the maximum peak prominence.

**Figure 5:**
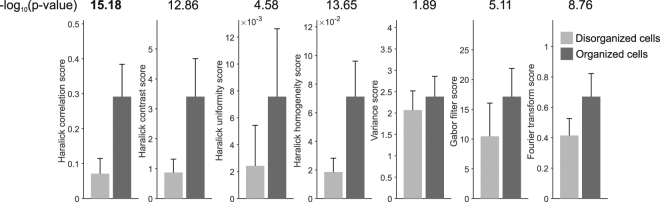
Comparison of sarcomere organization metrics in discriminating organized and disorganized cardiomyocytes. Selected highly organized (n = 32) and disorganized (n = 26) neonatal mouse cardiomyocytes were used to quantitatively compare methods for measuring sarcomere organization. Out of 13 Haralick pixel-based measurements, 4 were identified as candidates for sarcomere organization measurements. −log_10_(p-values) are reported above each pair. P-values are calculated by two-sample t-tests. Error bars show standard deviation.

To assess the performance of SOTA as well as previously proposed metrics of sarcomere organization^13,21,24^, sets of neonatal mouse atrial or ventricular CMs with highly organized (n = 32) or disorganized (n = 26) sarcomeres were compared. Multiple variations of SOTA using different Haralick texture features were used, in addition to Gabor filters and Fourier transforms (Fig. 5). Gabor filters use sinusoidal plane waves at specified orientations multiplied by a Gaussian function to detect edges in images^25^. These filters were applied at multiple orientations and wavelengths, and the maximum periodic response magnitude was used as the metric. Fourier transforms are also used to convert an image to the frequency domain to assess the repeating sarcomere structure^21,24^.

Among these various methods, the SOTA method based on the Haralick correlation metric best distinguished between cells with highly organized and disorganized sarcomeres. This may be in part due to the method’s ability to analyze pixels within the cellular region alone. In comparison, Fourier transforms must be applied to either the bounding box of an image, or a sub-region of the cell. Analyzing the bounding box image introduces artifacts associated with cell shape, while selecting a sub-region of the cell would leave out information or introduce bias and be less amenable to automation.

### Most reprogrammed cardiomyocytes have lower sarcomere organization

**Figure 6:**
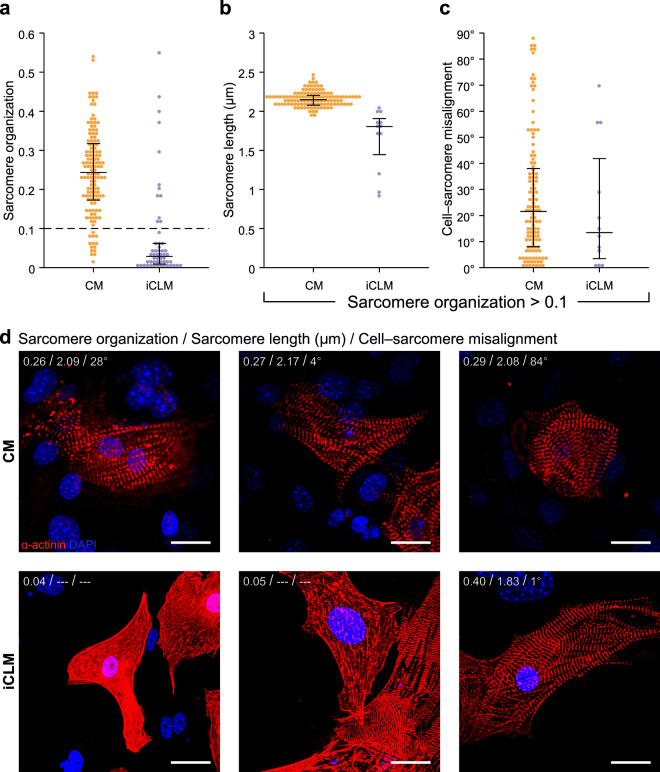
Sarcomere analysis of reprogrammed cardiomyocytes suggests an immature phenotype. (**a**) Sarcomere organization as calculated by Haralick correlation between neonatal mouse cardiomyocytes (CM) and fibroblast-reprogrammed induced cardiac-like myocytes (iCLM). Black bars represent 25^th^, 50^th^, and 75^th^ percentile. Only cells with sarcomere organization >0.1 were analyzed for (**b**) sarcomere length and (**c**) cell–sarcomere misalignment. (**d**) Example images of sarcomere metrics in CMs and iCLMs. Sarcomere length and cell–sarcomere misalignment were not reported if sarcomere organization score was below the 0.1 threshold. Scale bar = 20 μm.

We next used SOTA to compare sarcomere organization in reprogrammed iCLMs and endogenous CMs. Overall, sarcomere organization was markedly lower in iCLMs than in CMs, indicative of less mature cardiomyocytes produced by GHMT reprogamming (Fig. 6a). This result is consistent with our previous manual qualitative analysis, in which only ~20% of α-actinin^+^ cells were classifying as having visually distinguishable sarcomeres^12^. Though on average sarcomere organization was much lower in iCLMs, the presence of some iCLMs with highly organized sarcomeres indicates that this reprogramming method can produce cells on par with neonatal cardiomyocytes (Fig. 6d). iCLMs with highly organized sarcomeres were found in α-actinin^+^ cells, α-actinin^+^/Hcn4-GFP^+^ cells, and α-actinin^+^/Hcn4-GFP^+^/Nppa^+^ cells (Supplementary Figure S3). Sarcomere length was only accurately calculated for cells with sufficient sarcomere organization (sarcomere length >0.1). Cells with very low sarcomere organization scores would yield sarcomere length measurements well outside the normal range, possibly indicating other intensity-based features of the cells (Supplementary Figure S4). CMs with sufficient sarcomere organization for analysis had sarcomere lengths of about 2.2 μm, while iCLMs were typically lower, most of which fell in the 1.8–2.0 μm range (Fig. 6b). Smaller sarcomere lengths are typically found in immature cardiomyocytes, again pointing towards a less mature phenotype^26^. Several α-actinin^+^/Hcn4-GFP^+^ and α-actinin^+^/Nppa^+^ iCLMs had very low sarcomere lengths of about 1 μm, which were manually confirmed in ImageJ (Supplementary Figure S3). Surprisingly, these cells had very clear sarcomere structure, suggesting that this result may not be entirely due to an immature phenotype. Cell–sarcomere misalignment was also measured as the difference in angle between the orientation of the sarcomeres and the orientation of the major axis of the cell (Fig. 6c). A smaller difference in angle, in which the sarcomeres are oriented in the direction of the major axis, is observed in mature cardiomyocytes^27^. The cell–sarcomere misalignment was similar between CMs and iCLMs, however both cell types exhibited substantial variability. It should be noted that cells were not excluded based on cellular elongation. The orientation of the major axis in a less elongated cell is less meaningful, which may artificially result in a high misalignment score.

The various morphological and cytoskeletal metrics were compared to identify potential relationships between different developmental operations (Supplementary Figure S4). In the CMs, there was a slight upward trend in cellular elongation with increasing sarcomere organization, which was not observed in the iCLMs. Above the sarcomere organization threshold, CMs exhibited no relationship between sarcomere organization and sarcomere length, possibly indicating that sarcomere length is no longer a major indicator of further cell maturity. Below the sarcomere organization threshold, a highly variable sarcomere length was observed in both endogenous and reprogrammed cardiomyocytes. This threshold effect suggests that sarcomeric proteins must be assembled in a highly coordinated fashion to tightly regulate the characteristic sarcomere length of a given cardiomyocyte. Cell–sarcomere misalignment was lower in the more elongated CMs, a relationship not seen in the iCLMs. This is possibly due to a coordinated effort in endogenous cardiomyocyte development to place new sarcomeres along the leading edge of the cell^26^.

## Discussion

These methods introduce a new framework for using multiple immunofluorescence channels to automatically segment cells and analyze both morphological and cytoskeletal features of neonatal mouse and fibroblast-reprogrammed cardiomyocytes. The segmentation algorithm can be used without prior manual cell type classification, as it was with the reprogrammed cardiomyocytes. This allows for the identification of cells with any combination of cardiomyocyte markers, including the unexpected combinations we observed that are not seen *in vivo*.

Using our segmentation method, we found cells expressing cardiomyocyte markers similar to those of atrial cardiomyocytes (α-actinin^+^/Nppa^+^) and pacemaker cells (α-actinin^+^/Hcn4-GFP^+^). We have previously assessed GHMT-reprogrammed fibroblasts by patch-clamping^12^, and we found that induced atrial-like cells had action potentials similar to those of endogenous atrial cardiomyocytes. Similarly, induced pacemaker-like cells displayed action potentials resembling endogenous pacemaker cells. Although not assessed in this study, induced ventricular-like cells were also similar to endogenous ventricular cardiomyocytes. Morphologically, the α-actinin^+^/Hcn4-GFP^+^ cells resembled endogenous pacemaker cells in eccentricity and circularity, possibly suggesting a mature phenotype. To address the possibility that α-actinin^+^/Nppa^+^ iCLMs represented hypertrophic ventricular cardiomyocytes rather than atrial cardiomyocytes^28^, we compared the α-actinin^+^/Nppa^+^ iCLMs to the α-actinin^+^ cells, which may represent ventricular-like myocytes, and found no difference in cell size. Similarly, the α-actinin^+^/Nppa^+^ iCLMs are comparable in cell size to endogenous atrial cardiomyocytes. Although Hcn4 is dynamically expressed during heart development^17^, we have previously shown that these cells do not originate from an Nkx2.5^+^ progenitor cell type or from actively dividing cells, suggesting that these incompletely reprogrammed cells arise from a committed lineage^12^.

Previous methods of assessing sarcomere structure are limited by the range of orientations and spatial patterns studied^13^ or require manual intervention^21,24,29,30^. Furthermore, our previous analysis of cardiomyocyte induction by GHMT relied on categorical classification rather than a continuous variable^12^. The high-throughput methods introduced here are fully automated and generalized to quantify sarcomere organization regardless of cell shape, cell orientation, and image magnification. We accomplish this by measuring Haralick correlation values at a wide range of orientations and pixel offsets. Non-regular geometries are tolerated because the Haralick correlation metric can be computed using only pixels belonging to cells. Fourier transforms, which are more commonly used, are limited to rectangular images.

To our knowledge, automated sarcomere texture analysis has not previously been applied to reprogrammed cardiomyocytes. Here, we apply our methods to both neonatal cardiomyocytes and fibroblast-reprogrammed cardiomyocytes. Using the described methods, we found that few α-actinin^+^ iCLMs had organized sarcomeres (~22%), compared to most endogenous CMs (~89%). In addition, iCLMs had shorter sarcomere lengths on average, which are typically seen in immature cardiomyocytes. Further, we found that cell elongation increases with sarcomere organization in the CMs but not the iCLMs. This suggests there may be a higher-level coordination between cell shape and sarcomere organization that has not been addressed in previous reprogramming studies. Indeed, it has been shown that, when cultured on micropatterned plates, sarcomeres preferentially align with the major axis of elongated neonatal cardiomyocytes^27,31^.

Here we describe the utility of our algorithm for objectively evaluating cardiac reprogramming, but we envision that SOTA can be applied to additional research questions of considerable biological significance. For example, texture analysis could be similarly used to compare endogenous, reprogrammed, and iPS-derived cardiomyocytes, for which there remains concern about maturity and functionality^32^. Furthermore, SOTA could be used to track sarcomere formation in real-time in combination with appropriate fluorescent markers. Thus, we can foresee that such studies would allow more robust dissection of the molecular mechanisms that regulate sarcomere assembly^33^. From a more translational standpoint, real-time assessments of sarcomere formation could inform future screening efforts to optimize generation of functional cardiomyocytes. We also envision that our texture analysis approach could be applied to other muscle types, such as skeletal and smooth muscle, or even to other structurally complex cell types, such as neurons.

Several recent studies have described elegant and innovative whole-transcriptome approaches to characterize the potential functionality of specific cell types^34,35^. However, we propose that gene expression signatures alone are unlikely to characterize the full range of intricately coordinated processes required for generating functional cardiomyocyte subtypes. For example, sarcomere gene expression does not guarantee efficient assembly and organization. It is likely that a combination of sarcomere protein stoichiometry, chaperone proteins, and specific post-translational modifications are required for proper sarcomere organization in addition to sarcomere gene expression. Based on the potential importance of these non-transcriptional mechanisms, we believe that the sarcomere organization metrics described in our study will provide crucial information that remains uncaptured by current whole-transcriptome approaches. It is likely that combining the sarcomere analysis method with other approaches, such as electrophysiological and contractility measurements, will ultimately be required to functionally optimize cellular engineering approaches for potential clinical translation.

## Methods

### Isolation of mouse fibroblasts

All experimental procedures involving animals were approved by the Institutional Animal Care and Use Committee at UT Southwestern Medical Center. All experiments and methods were performed in accordance with relevant guidelines and regulations. Mouse embryonic fibroblasts (MEFs) were isolated from E13.5 mouse embryos of Hcn4-GFP reporter or wild-type mice. The embryos were separated from the placenta and surrounding membranes. The head and the internal organs of the chest and abdominal cavities were removed from the embryos. The remaining tissues were minced and digested with 0.25% trypsin for 15 min at 37 °C to obtain single-cell suspensions. The isolated cells were cultured in fibroblast medium containing 10% FBS and 1% penicillin/streptomycin. These cells were trypsinized and replated the next day. Adult tail-tip fibroblasts (TTFs) were isolated using explant culture as described previously^8^. Hearts from adult Hcn4-GFP mice were minced into small pieces which were cultured in fibroblast medium. The medium was changed every 2−3 days. After ~10 days in culture, adult cardiac fibroblasts were harvested.

### Isolation of CMs

Neonatal mouse ventricular CMs were isolated using the Neomyts kit (Cellutron) as per manufacturer’s protocol. Neonatal atrial and pacemaker CMs were isolated using methods modified from Sreejit *et al*.^36^. P0−P1 hearts were dissected, washed in ice-cold PBS, and placed in Cold Balanced Solution (20 mmol/L HEPES 7.6, 130 mmol/L NaCl, 1 mmol/L NaH2PO4, 4 mmol/L glucose, and 3 mmol/L KCl). The right atrium was manually dissected and minced extensively in a minimal volume of 0.05% trypsin. Atrial tissue was incubated with 0.25% trypsin and agitated for 4 min in a 37 °C shaking water bath before allowing tissue to settle for 1 min without agitation. The first fraction was collected by removing the removing the supernatant to a fresh tube containing Culture Medium (DMEM, 20% FBS, 2 mmol/L L-glutamine, and 3 mmol/L sodium pyruvate). This cycle was repeated 3 times to collect a total of 4 fractions that were pooled, passed through a 100 μm filter, and combined with additional Culture Medium before plating on glass coverslips that had been previously coated with 2% gelatin for at least 10 min. After initial plating, the medium was changed after 72 h, and again every 48 h thereafter.

### iCLM reprogramming

Generation of retroviral constructs of mouse Gata4, Hand2, Mef2c, and Tbx5 was performed as previously described^8^. Retroviral constructs were transfected using Fugene 6 (Promega) into Platinum E cells (Cell Biolabs). 24 h after transfection, the viral medium (the media cultured with Platinum E cells) was collected and polybrene was added to viral medium that was filtered through a 0.45 μm filter at a concentration of 6 μg/μL. The mixture replaced the growth medium in the cell culture plate with mouse fibroblasts. Platinum E cells were replenished with the growth medium (DMEM with 10% FBS). 24 h later, mouse fibroblasts were re-infected with the second viral medium from Platinum E cell plate as described above for the first infection. Another 24 h later, viral medium on the plate with mouse fibroblasts was replaced with induction medium, composed of DMEM/199 (4:1), 10% FBS, 5% horse serum, 1% penicillin/streptomycin, 1% non-essential amino acids, 1% essential amino acids, 1% B-27, 1% insulin-selenium-transferrin, 1% vitamin mixture, and 1% sodium pyruvate (Invitrogen). 10% conditioned medium obtained from rat neonatal cardiomyocyte culture in DMEM/199 (4:1) with 5% FBS as described previously^8^. Conditioned medium was filtered through a 0.22 μm filter. This medium was changed every 2–3 days until cells were harvested.

### Immunocytochemistry

Cells were fixed with 4% paraformaldehyde for 15 min and permeabilized with permeabilization buffer (0.05% Triton-X in PBS) for 5 min three times at room temperature. Cells were blocked with blocking buffer (Universal blocking buffer, BiogeneX) for 30 min and then incubated with primary antibodies against cTnT (Mouse monoclonal, Thermo Scientific, 1:400), α-actinin (Mouse monoclonal, Sigma, 1:400 dilution), GFP (Chicken IgY fraction, Invitrogen, 1:400 dilution), Nppa (Rabbit polyclonal, Abgent, 1:200 dilution), Myl2 (Rabbit polyclonal, Protein tech, 1:200 dilution), Myl7 (Rabbit polyclonal, Protein tech, 1:200 dilution), Myl7 (Mouse monoclonal, Synaptic system, 1:200 dilution), Hcn4 (Mouse monoclonal, NeuroMab, 1:200 dilution), PCM1 (Rabbit polyclonal, Sigma, 1:200 dilution) for 1 hr at room temperature or overnight at 4 °C (for Myl2, Myl7, Hcn4, and PCM1 antibodies). Following washing three times for 5 min with permeabilization buffer, cells were incubated with appropriate Alexa fluorogenic secondary antibodies (Invitrogen or Abcam) to detect the signal at room temperature for 1 hr. After another set of washing (5 min ×3 with permeabilization buffer), cells were mounted with Vectashield with DAPI and images were captured with Zeiss LSM 500 confocal microscope.

### Cell segmentation algorithm

Image analysis was performed on images from reprogrammed cells acquired in a previous study^12^ as well as images from new experiments using the same GHMT reprogramming methods and imaged as described above. Due to bleed-through of the Nppa and Hcn4-GFP channels into the DAPI channel, DAPI images were corrected by assigning pixels with >1.5 DAPI intensity to (NPPA or Hcn4-GFP) intensity ratio to zero. DAPI images were then smoothed using morphological closing followed by a Gaussian filter with a radius of 4 pixels. The image was then binarized using an Otsu threshold, which maximizes the variance between foreground and background pixel intensities. Small objects were removed from the image and nuclei were assigned an object number.

The Hcn4-GFP and α-actinin channels were similarly filtered with a Gaussian blur, and Otsu thresholding was done to produce binary images. The nuclei objects were overlaid with the Hcn4-GFP or α-actinin channel. Objects that were fully enclosed within the foreground of the image were classified as Hcn4^+^ or α-actinin^+^. If the objects were fully within the foreground of the Hcn4-GFP/α-actinin intersection, they were categorized as Hcn4-GFP^+^/α-actinin^+^. Shortest distances were calculated between same-class nuclei, and nuclei within 25 pixels (~3.9 μm) of one another were treated as binucleates. Object numbers were subsequently reassigned.

To determine cell boundaries, α-actinin^+^ cells and Hcn4-GFP^+^ cells were segmented first, in parallel. The Hcn4-GFP binary image was used to mask the α-actinin image and likewise the α-actinin binary image was used to mask the Hcn4-GFP image. Cells were segmented and these cellular regions were converted to masks to be applied to the combined Hcn4-GFP/α-actinin image. α-actinin^+^/Hcn4-GFP^+^ cells were then segmented in the masked image. In all cases, watershed segmentation was done on the gradient transformed image to determine cell boundaries^15^. Sobel horizontal and vertical edge-emphasizing filters were applied to the image and the magnitude of the two filtered images was taken. The marker-controlled watershed segmentation algorithm was used, treating nuclei as internal markers.

Because Nppa is perinuclear, it was not expected that Nppa signal would be high within the nucleus. Therefore, to classify cell objects for Nppa, a perinuclear ring was created for each nucleus. The perinuclear ring was defined as the area extending 8 pixels (1.25 μm) radially from the edge of the nucleus. Nppa intensity was measured in this region. If the 90^th^ percentile intensity was greater than a manually determined threshold of 0.1 (relative intensity), cells were classified as Nppa^+^.

Cell segmentation and sarcomere organization algorithms were written in MATLAB. A slightly modified cell segmentation algorithm was also developed in CellProfiler^37^. This version uses manually determined mean intensity thresholding in the nuclear area to classify cells instead of whole-image thresholding.

### Cell morphology and sarcomere organization metrics

MATLAB’s image processing toolbox was used to compute cell size and shape characteristics. Cell area was computed in pixels and converted to μm^2^. Cell elongation was calculated as the ratio of the major axis length to the minor axis length. Cell circularity is equal to $4\pi \times Area\times Perimete{r}^{-2}$, with a value of 1 indicating a perfect circle. Cell eccentricity specifies the eccentricity of the ellipse with the same second-moments as the cellular region. Eccentricity of the ellipse is calculated as the ratio of the distance between the foci and the major axis length. Cell eccentricity is 0 for a perfect circle.

Sarcomere organization is calculated using Haralick features, which are pixel intensity-based algorithms for quantifying image texture^22,23^. First, a gray-level co-occurrence matrix is calculated for given orientation and pixel pair offset distances. The co-occurrence matrix p calculates the frequency at which pixels within a specified intensity range are matched by spatially separated pixels of the same intensity. The result is a *g* × *g* matrix, where g is the number of gray-levels (or intensity bins) that are to be considered. The default value was used, which for grayscale images is 8 (and 2 for binary images). From the co-occurrence matrix, 13 texture features can be measured. Haralick correlation is one of these features that measures the likelihood of finding two pixels of similar intensity separated by a given distance. The correlation value is calculated by the Equation 1:

1 $$\begin{array}{ccc}{\rm{H}}{\rm{a}}{\rm{r}}{\rm{a}}{\rm{l}}{\rm{i}}{\rm{c}}{\rm{k}}\,{\rm{c}}{\rm{o}}{\rm{r}}{\rm{r}}{\rm{e}}{\rm{l}}{\rm{a}}{\rm{t}}{\rm{i}}{\rm{o}}{\rm{n}} & = & \sum _{i,j}\frac{(i-{\mu }_{i})(j-{\mu }_{j})p(i,j)}{{\sigma }_{i}{\sigma }_{j}}\\ {\mu }_{i}=\sum _{i}i{p}_{i}(i) &  & {\mu }_{j}=\sum _{j}j{p}_{j}(j)\\ {\sigma }_{i}=\sqrt{\sum _{i}{(i-{\mu }_{i})}^{2}{p}_{i}(i)}\, &  & {\sigma }_{j}=\sqrt{\sum _{j}{(j-{\mu }_{j})}^{2}{p}_{j}(j)}\end{array}$$

p(i,j) is the *i*th row and *j*th column of the co-occurrence matrix, and *p*_*i*_ and *p*_*j*_ are the marginal probabilities of the co-occurrence matrix.

Calculation of the co-occurrence matrix and corresponding Haralick correlation values was repeated at many orientation angles and spatial distances, resulting in an m×n matrix of Haralick correlation values, where m is the number of angles (values are symmetric about 180°) and n is the number of pixel offsets. We then used MATLAB’s *interp1* function on each row to achieve sub-pixel resolution. Visualization of this data can be seen in Fig. 3.

Other Haralick features that were considered in the comparison analysis include contrast (Equation 2), uniformity (Equation 3, also known as energy or angular second moment), and homogeneity (Equation 4), calculated from the co-occurrence matrix as follows:

2 $${\rm{H}}{\rm{a}}{\rm{r}}{\rm{a}}{\rm{l}}{\rm{i}}{\rm{c}}{\rm{k}}\,{\rm{c}}{\rm{o}}{\rm{n}}{\rm{t}}{\rm{r}}{\rm{a}}{\rm{s}}{\rm{t}}=\sum _{i,j}{|i-j|}^{2}p(i,j)$$

3 $${\rm{H}}{\rm{a}}{\rm{r}}{\rm{a}}{\rm{l}}{\rm{i}}{\rm{c}}{\rm{k}}\,{\rm{u}}{\rm{n}}{\rm{i}}{\rm{f}}{\rm{o}}{\rm{r}}{\rm{m}}{\rm{i}}{\rm{t}}{\rm{y}}=\sum _{i,j}p{(i,j)}^{2}$$

4 $${\rm{H}}{\rm{a}}{\rm{r}}{\rm{a}}{\rm{l}}{\rm{i}}{\rm{c}}{\rm{k}}\,{\rm{h}}{\rm{o}}{\rm{m}}{\rm{o}}{\rm{g}}{\rm{e}}{\rm{n}}{\rm{e}}{\rm{i}}{\rm{t}}{\rm{y}}=\sum _{i,j}\frac{p(i,j)}{1+|i-j|}$$

We also measured a variance value, which is the sum of the Haralick contrast values at an offset distance of 1 pixel. We expected the variance value to be close to zero for homogenous images and close to one for patterned images.

Gabor filters were applied at a variety of spatial frequencies and orientations using MATLAB’s *imgaborfilt* function. The response magnitudes were normalized to cell area. Magnitudes were then plotted against wavelength for each orientation. Each profile was fitted to the sum of a quadratic function and a Gaussian function using MATLAB’s *lsqnonlin* function to identify aperiodic and periodic components. A quadratic function was chosen instead of an exponential function because it appeared to fit better at longer wavelengths. The maximum amplitude of the Gaussian function was used as the Gabor filter score.

To generate Fourier transform scores, 2D Fast Fourier transforms were applied to the bounding box of the α-actinin channel, which produced a transformed image of the same dimensions. The subsequent analysis was done in a manner similar to that of a previously described method^21,24^. The transformed image was radially integrated along 360 dimensions to generate a frequency profile. This profile was fit to the sum of two exponential functions and one Gaussian function using MATLAB’s *lsqnonlin* function to identify aperiodic and periodic components. Sarcomere organization was calculated as the area under the Gaussian curve.

### Data availability

The datasets generated during and/or analyzed during the current study are available from the corresponding author on reasonable request. The MATLAB code and CellProfiler files used for the analysis are available at http://bme.virginia.edu/saucerman/.

## Electronic supplementary material

Supplementary information

## References

[1] Tzahor E, Poss KD (2017). Cardiac regeneration strategies: Staying young at heart. Science.

[2] Prabhu SD, Frangogiannis NG (2016). The Biological Basis for Cardiac Repair After Myocardial Infarction: From Inflammation to Fibrosis. Circ. Res..

[3] Ma Y, Iyer RP, Jung M, Czubryt MP, Lindsey ML (2017). Cardiac Fibroblast Activation Post-Myocardial Infarction: Current Knowledge Gaps. Trends Pharmacol. Sci..

[4] Shiba Y (2012). Human ES-cell-derived cardiomyocytes electrically couple and suppress arrhythmias in injured hearts. Nature.

[5] Chong JJH (2014). Human embryonic-stem-cell-derived cardiomyocytes regenerate non-human primate hearts. Nature.

[6] Sadahiro T, Yamanaka S, Ieda M (2015). Direct cardiac reprogramming: progress and challenges in basic biology and clinical applications. Circ. Res..

[7] Srivastava D, DeWitt N (2016). *In Vivo* Cellular Reprogramming: The Next Generation. Cell.

[8] Song K (2012). Heart repair by reprogramming non-myocytes with cardiac transcription factors. Nature.

[9] Qian L (2012). *In vivo* reprogramming of murine cardiac fibroblasts into induced cardiomyocytes. Nature.

[10] Ieda M (2010). Direct reprogramming of fibroblasts into functional cardiomyocytes by defined factors. Cell.

[11] Inagawa K (2012). Induction of cardiomyocyte-like cells in infarct hearts by gene transfer of Gata4, Mef2c, and Tbx5. Circ. Res..

[12] Nam Y-J (2014). Induction of diverse cardiac cell types by reprogramming fibroblasts with cardiac transcription factors. Dev. Camb. Engl..

[13] Bass GT (2012). Automated image analysis identifies signaling pathways regulating distinct signatures of cardiac myocyte hypertrophy. J. Mol. Cell. Cardiol..

[14] Otsu N (1979). A Threshold Selection Method from Gray-Level Histograms. IEEE Trans. Syst. Man Cybern..

[15] Vincent L, Soille P (1991). Watersheds in digital spaces: an efficient algorithm based on immersion simulations. IEEE Trans. Pattern Anal. Mach. Intell..

[16] Zebrowski DC (2015). Developmental alterations in centrosome integrity contribute to the post-mitotic state of mammalian cardiomyocytes. eLife.

[17] Liang X (2013). HCN4 dynamically marks the first heart field and conduction system precursors. Circ. Res..

[18] Zúñiga R, González D, Valenzuela C, Brown N, Zúñiga L (2016). Expression and cellular localization of HCN channels in rat cerebellar granule neurons. Biochem. Biophys. Res. Commun..

[19] Wen Y, Li B (2015). Morphology of mouse sinoatrial node and its expression of NF-160 and HCN4. Int. J. Clin. Exp. Med..

[20] Abad M (2017). Notch Inhibition Enhances Cardiac Reprogramming by Increasing MEF2C Transcriptional Activity. Stem Cell Rep..

[21] Pasqualini FS, Sheehy SP, Agarwal A, Aratyn-Schaus Y, Parker KK (2015). Structural phenotyping of stem cell-derived cardiomyocytes. Stem Cell Rep..

[22] Haralick RM, Shanmugam K, Dinstein I (1973). Textural Features for Image Classification. IEEE Trans. Syst. Man Cybern..

[23] Haralick, R. M. & Shapiro, L. G. *Computer and Robot Vision*. (Addison-Wesley Longman Publishing Co., Inc., 1992).

[24] Sheehy SP (2014). Quality metrics for stem cell-derived cardiac myocytes. Stem Cell Rep..

[25] Fogel I, Sagi D (1989). Gabor filters as texture discriminator. Biol. Cybern..

[26] Yang X, Pabon L, Murry CE (2014). Engineering adolescence: maturation of human pluripotent stem cell-derived cardiomyocytes. Circ. Res..

[27] Bray M-A, Sheehy SP, Parker KK (2008). Sarcomere alignment is regulated by myocyte shape. Cell Motil. Cytoskeleton.

[28] Shubeita HE (1990). Endothelin induction of inositol phospholipid hydrolysis, sarcomere assembly, and cardiac gene expression in ventricular myocytes. A paracrine mechanism for myocardial cell hypertrophy. J. Biol. Chem..

[29] Aoki H, Sadoshima J, Izumo S (2000). Myosin light chain kinase mediates sarcomere organization during cardiac hypertrophy *in vitro*. Nat. Med..

[30] Chopra A, Patel A, Shieh AC, Janmey PA, Kresh JY (2012). α-Catenin localization and sarcomere self-organization on N-cadherin adhesive patterns are myocyte contractility driven. PloS One.

[31] Kuo P-L (2012). Myocyte shape regulates lateral registry of sarcomeres and contractility. Am. J. Pathol..

[32] Yoshida Y, Yamanaka S (2017). Induced Pluripotent Stem Cells 10 Years Later: For Cardiac Applications. Circ. Res..

[33] Sparrow JC, Schöck F (2009). The initial steps of myofibril assembly: integrins pave the way. Nat. Rev. Mol. Cell Biol..

[34] Zhang Y (2015). Epigenomic Reprogramming of Adult Cardiomyocyte-Derived Cardiac Progenitor Cells. Sci. Rep..

[35] Cao N (2016). Conversion of human fibroblasts into functional cardiomyocytes by small molecules. Science.

[36] Sreejit P, Kumar S, Verma RS (2008). An improved protocol for primary culture of cardiomyocyte from neonatal mice. In Vitro Cell. Dev. Biol. Anim..

[37] Carpenter AE (2006). CellProfiler: image analysis software for identifying and quantifying cell phenotypes. Genome Biol..

