# Supplementary material for: High content analysis identifies unique morphological features of reprogrammed cardiomyocytes
Source: Sci Rep. 2018 Jan 19;8:1258. doi: 10.1038/s41598-018-19539-z (PMC5775342; doi:10.1038/s41598-018-19539-z)
Supplement: Supplementary file 1 — Supplementary information [file 41598_2018_19539_MOESM1_ESM.pdf]

Supplementary information

High content analysis identifies unique morphological features of reprogrammed cardiomyocytes

Matthew D. Sutcliffe[1], Philip M. Tan[1], Antonio Fernandez-Perez[2], Young-Jae Nam[3], Nikhil V. Munshi[2,4,5,6], Jeffrey J. Saucerman[1]*

[1] Department of Biomedical Engineering, University of Virginia, Charlottesville, VA 22908, USA
[2] Department of Internal Medicine, Division of Cardiology, UT Southwestern Medical Center, Dallas, TX 75390, USA
[3] Department of Medicine, Division of Cardiovascular Medicine, Vanderbilt University Medical Center, Nashville, TN 37232, USA
[4] Department of Molecular Biology, UT Southwestern Medical Center, Dallas, TX 75390, USA
[5] McDermott Center for Human Growth and Development, UT Southwestern Medical Center, Dallas, TX 75390, USA
[6] Hamon Center for Regenerative Science and Medicine, UT Southwestern Medical Center, Dallas, TX 75390, USA

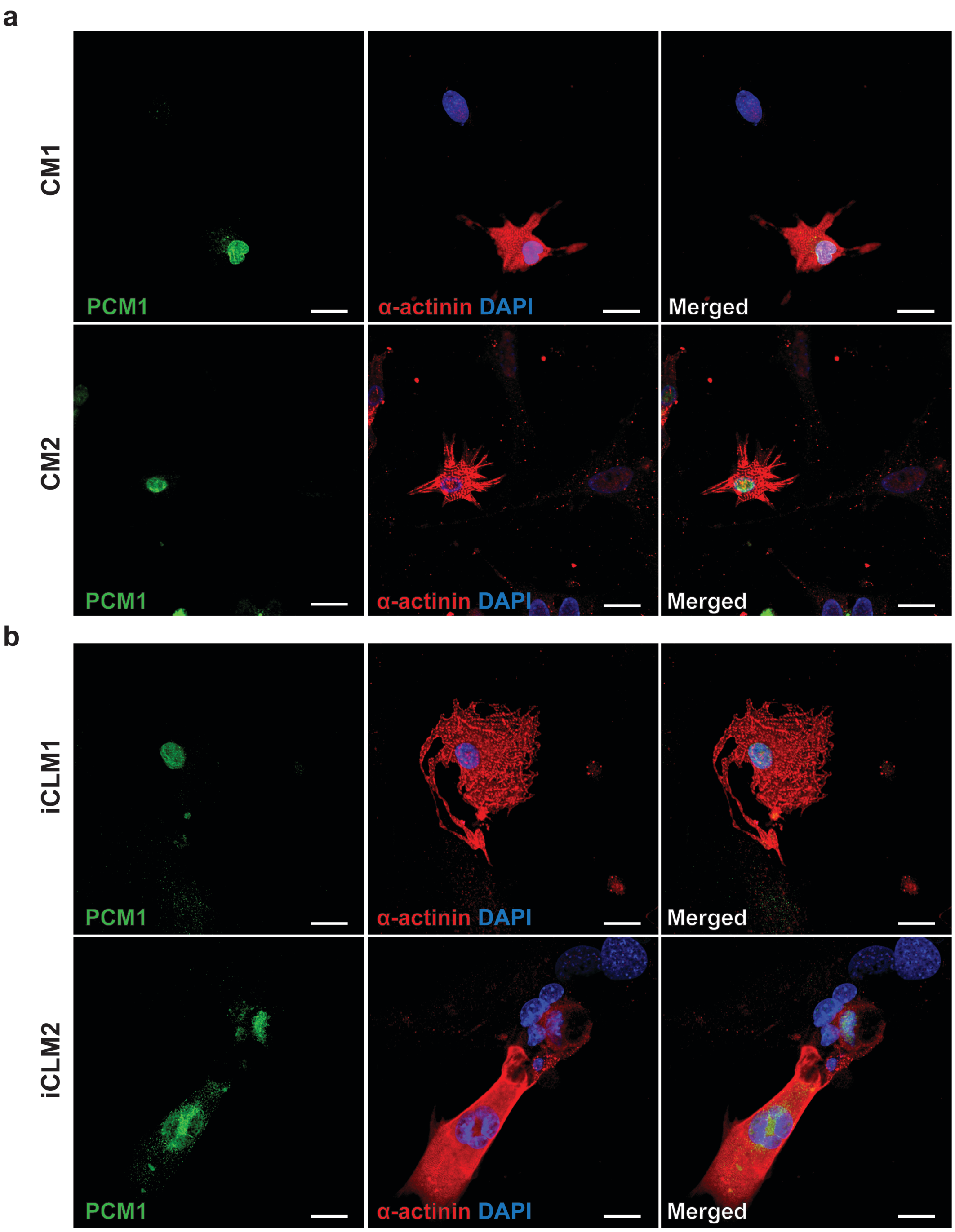

Supplementary Figure S1. Confirmation of cardiomyocyte induction and maturity by PCM1 staining. Representative (a) endogenous CMs and (b) reprogrammed iCLMs both express pericentriolar material 1 (PCM1) in the nuclear and perinuclear regions, suggesting mature cardiomyocyte induction[1,2]. In mitotic cells, PCM1 is found throughout the cytoplasm, associating with centrosomes during the cell cycle. PCM1 re-localizes to the nuclear envelope in post-mitotic cells, and this localization would be expected in terminally differentiated cardiomyocytes. Scale bar = 20 μm.

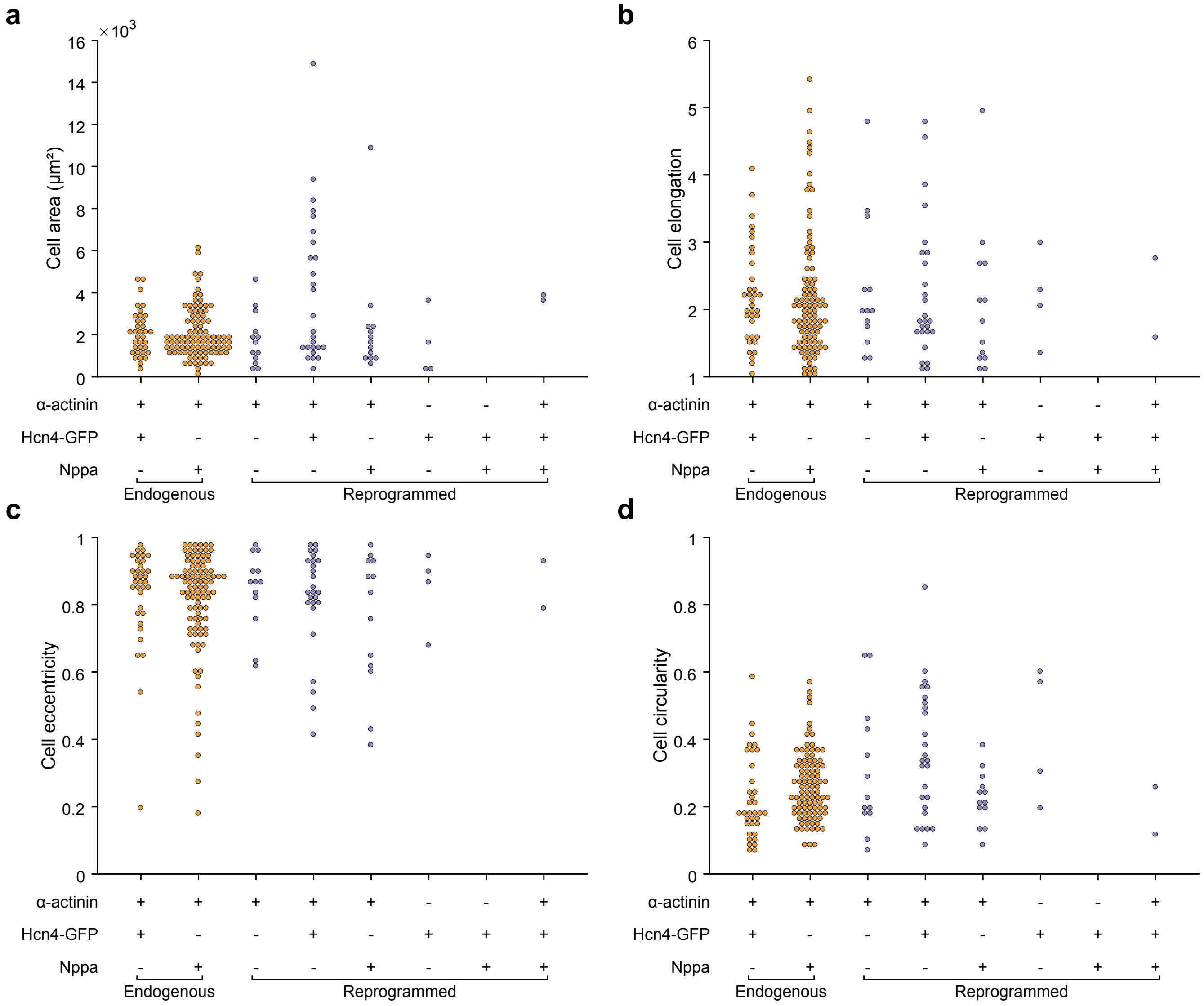

Supplementary Figure S2. Cell morphology metrics by subtype. (a) Most of the variability in iCLM cell area is seen in α-actinin⁺/Hcn4-GFP⁺ cells. The variability in (b) cell elongation, (c) cell eccentricity, and (d) cell circularity, is spread out equally among the different subtypes.

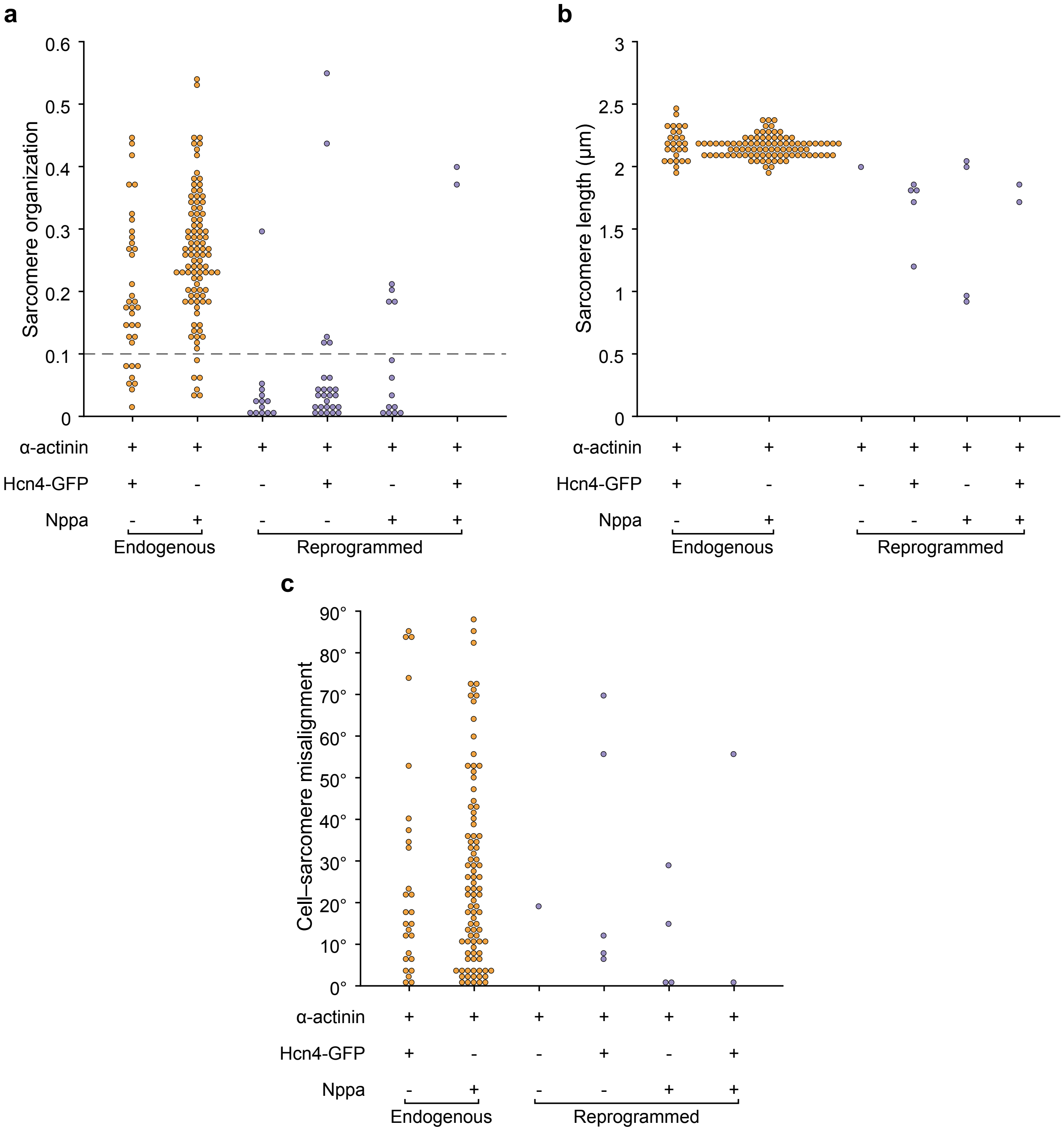

Supplementary Figure S3. Sarcomere analysis metrics by subtype. (a) All iCLM subtypes were able to produce cells with sarcomere organization > 0.1. (b) iCLMs with very low sarcomere lengths were either α-actinin⁺/Hcn4-GFP⁺ or α-actinin⁺/Nppa⁺. (c) No clear pattern in cell−sarcomere misalignment among iCLM subtypes.

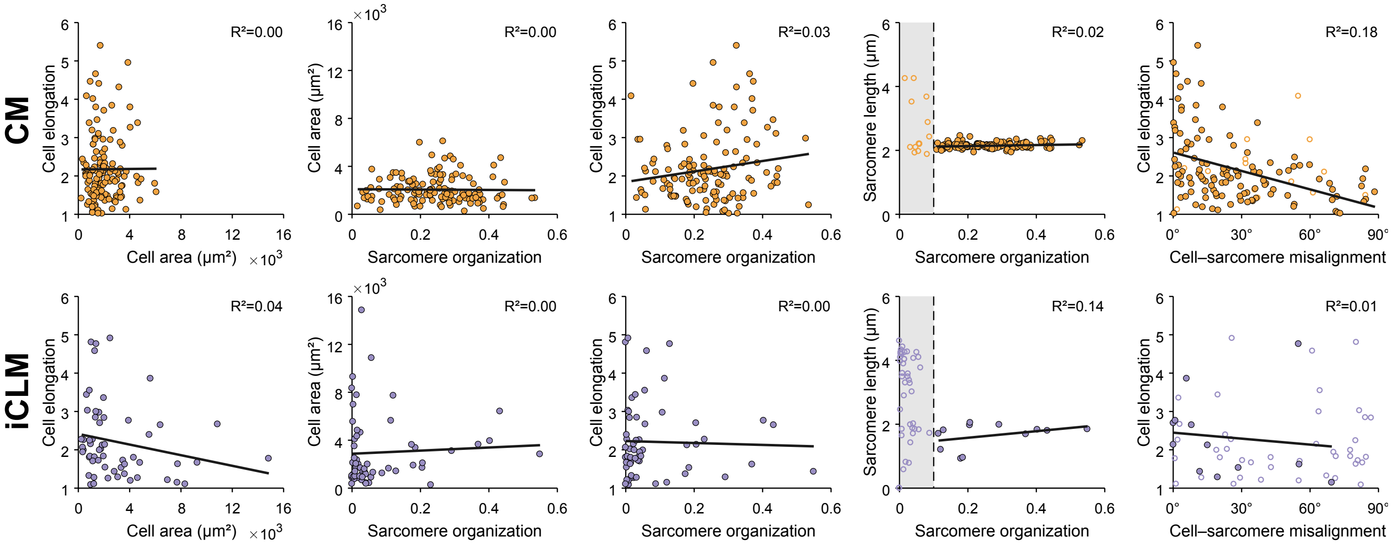

Supplementary Figure S4. Scatter plots of cell morphology relationships. CMs (top) and iCLMs (bottom) were measured for cell morphology and sarcomere organization. Open circles represent cells with below-threshold sarcomere organization.

References

1. Zebrowski, D. C. *et al.* Developmental alterations in centrosome integrity contribute to the post-mitotic state of mammalian cardiomyocytes. *eLife* **4,** e05563 (2015).
2. Bergmann, O. *et al.* Identification of cardiomyocyte nuclei and assessment of ploidy for the analysis of cell turnover. *Exp. Cell Res.* **317,** 188–194 (2011).
